# Bacteria as computers making computers

**DOI:** 10.1111/j.1574-6976.2008.00137.x

**Published:** 2008-11-11

**Authors:** Antoine Danchin

**Affiliations:** Génétique des Génomes Bactériens, Institut PasteurParis, France

**Keywords:** minimal genome, operating system, algorithmic complexity, junk DNA, APOBEC, ADAR

## Abstract

Various efforts to integrate biological knowledge into networks of interactions have produced a lively microbial systems biology. Putting molecular biology and computer sciences in perspective, we review another trend in systems biology, in which recursivity and information replace the usual concepts of differential equations, feedback and feedforward loops and the like. Noting that the processes of gene expression separate the genome from the cell machinery, we analyse the role of the separation between machine and program in computers. However, computers do not make computers. For cells to make cells requires a specific organization of the genetic program, which we investigate using available knowledge. Microbial genomes are organized into a paleome (the name emphasizes the role of the corresponding functions from the time of the origin of life), comprising a constructor and a replicator, and a cenome (emphasizing community-relevant genes), made up of genes that permit life in a particular context. The cell duplication process supposes rejuvenation of the machine and replication of the program. The paleome also possesses genes that enable information to accumulate in a ratchet-like process down the generations. The systems biology must include the dynamics of information creation in its future developments.

## Introduction

‘Systems Biology’ is a fashionable domain in biological science. But do we have a precise idea of what the field covers? An answer may come from the observation that most definitions of the systems biology are related to explicit research programmes, which all emphasize the present need to integrate the considerable amount of knowledge that has accumulated in biology over the past 50 years or so (Bruggeman & Westerhoff, 2007; Laub *et al.*, 2007; Marles-Wright & Lewis, 2007; Rokem *et al.*, 2007; Bingle *et al.*, 2008; Potvin *et al.*, 2008). Many paths can be followed in the pursuit of the aim of integration, and I choose to review here a slightly unusual one, that of considering the cell as a computer making computers. Having revisited the history and the concepts of molecular biology with this aim in focus, I follow the path opened up by the pioneering investigators who took seriously what was (and usually still is) just perceived as a metaphor, the concept of the *genetic program*. Using a variety of sources, I show that a cell can be seen as a computer (a machine expressing a program), and review the evidence in support of the cell having the properties required to reproduce the computing machine while replicating its program. This view takes into account the important paradox raised by the obvious observation that computers do not make computers (yet). It provides an entry point for the category of *information* as a fundamental category of nature that all future developments of systems biology need to include (Danchin, 2008a).

To set the stage with a historical view of what could be a central paradigm permitting the success of systems biology, let us quote a paragraph from the presentation of the topic by the Institute for Systems Biology created at the turn of the millenium by Leroy Hood in Seattle: ‘Systems biology emerged as the result of the genetics “catalog” provided by the Human Genome project, and a growing understanding of how genes and their resulting proteins give rise to biological form and function. The study of systems biology has been aided by the ease with which the internet allows researchers to store and distribute massive amounts of information, plus advances in powerful new research technologies, and the infusion of scientists from other disciplines, e.g. computer scientists, mathematicians, physicists, and engineers.’ Systems biology, then, begins with inventories, and develops as an interdisciplinary science. This latter adjective is another fashionable word that underscores the importance of an intimate association between the concepts and technologies underlying widely separated areas of science – biochemistry, genetics and computer science. The statement also points out the importance of information, and this justifies investigating in some depth the present status of information theories.

Historically, systems biology follows on from molecular biology, a science based on many concepts more closely linked to arithmetic and computation than to classical physics or chemistry. Molecular biology relies heavily on concepts such as ‘control’, ‘coding’ or ‘information’, which are at the heart of arithmetic and computation. To accept the cell as a computer conjecture first requires an exploration of the concept of information, in relation to the concept of genetic program. Systems biology being highly multidisciplinary, this article has the difficult task of helping microbiologists become familiar with some unexpected developments in genomics, which are rooted in very abstract regions of knowledge, namely Number Theory. However, at some point, we need to leave the world of abstraction to come back to more mundane biology, via the exploration of the structure of genomes (essentially bacterial genomes, here), to link abstraction with the concrete world of metabolites, proteins, genes and cells. We devote a significant part of our review of the literature to the task of pinning down the relationship between the abstract domain of information and the concrete domain of its creation and management in the cell.

Why is this emphasis on information so important? In addition to his seminal role in computer sciences, Alan Turing, a central figure in the conceptualization of information, was also responsible for many of the ideas used today in biology, both through his theory of growth and biological forms (Turing, 1952), and through his theory of computation [Turing, 1936–1937, 1946 (1986)]. Even at a fairly popular level, the involvement of information and Number Theory in biology is not new. It has been developed extensively by Douglas Hofstadter in a famous book, *Gödel, Escher, Bach, an Eternal Golden Braid*, which won the Pulitzer Prize in 1979. But how many people really understand that strings of symbols – such as those found in the sequence of DNA – can produce unexpected (emergent) outcomes when they are associated with a coding process (Hofstadter, 1979)? The Austrian mathematician Kurt Gödel showed that arithmetic (the science of whole numbers) can make statements about itself. To substantiate this remarkable claim, which implies that just manipulating whole numbers with the rules of arithmetic can generate novel information, Gödel used a simple trick. He coded the words used in Number Theory as integers (e.g. four, which is *quatre* in French, *vier* in German and τɛσσɛρα in Greek, can be coded by *4*) and used the corresponding code to translate propositions of arithmetic. This generated a large whole number, which could be manipulated by the rules of arithmetic, and after a sequence of operations, this manipulation generated another whole number. The latter could be decoded using the initial code. Gödel's trick was to drive the sequence of operations modifying the initial statement, to lead to a very particular conclusion. When decoded, the manipulated sequence translated into a particular proposition, which, briefly, stated: ‘I am impossible to prove’. In other words, arithmetic is incomplete, i.e. some propositions of arithmetic can be understood as valid; yet they cannot be proven within the frame of arithmetic. But this ‘incompleteness’ can also be seen as a positive feature; it is what allows the creation of new information – in Gödel's case, the statement of a fact of which the world was previously unaware. In his book, Hofstadter showed that the genetic code, which enables the world of nucleic acids to be translated into the world of proteins, which in turn manipulate nucleic acids, behaves exactly as Gödel's code does. This implies that manipulating strings of symbols, via a process that uses a code, can generate novel information. Of course, in the case of nucleic acids and proteins, there is no Gödel to drive the process, and no need for one: while Gödel knew what he was aiming at, living systems will accumulate information through recursivity, without any design being required. We only perceive a design because the end result is familiar to us, and thus seems more ‘right’ than any other possible result. But what we commonly term the ‘genetic program’ because it unfolds through time in a consistent manner is not a programme with an aim – it is merely there, and functions because it cannot do otherwise.

This observation, that the manipulation of strings of symbols can produce new information, may have considerable consequences in the development of new avenues for systems biology, and will be at the heart of the present review.

Despite the conceptual importance of this view, at present, few investigators would easily accept that there is more than a crude metaphor behind the analogy between cells and computers (see, however, Liberman, 1979; Yockey, 1992; Danchin, 1996; Liberman & Minina, 1996; Maynard-Smith, 2000). Yet the literature exploring the conjecture that the genetic program is more than a metaphor, and that cells, bacteria in particular, are Turing machines [i.e. behave as if they were computing devices (we shall not discuss here the nature of computing, save to say that it would be purely declarative, that is, not intentional, in a way similar to that proposed in lambda-calculus by Barendregt (1984))], provides an answer to many of the enigmas raised by the continuous production of information by living organisms. New forms, emerging structures and processes can be accounted for without having to rely on any novel or external principle (Danchin, 2003), and this can be the starting point for new families of experiments.

If there is something valid in the conjecture, then it must be taken into account when analysing the organization of genomes and the functions associated with genes, as well as the general features of evolution by natural selection (Quastler, 1953, 1964; Quastler *et al.*, 1958; Yockey *et al.*, 1958; Liberman, 1979; Yockey, 1992; Danchin, 1995, 1996, 2003, 2008a; Liberman & Minina, 1996; Danchin & Hénaut, 1997; Danchin *et al.*, 2000).

Much of the literature involved in this exploration does not appear in journals or books familiar to microbiologists, nor is it always indexed in PubMed (fortunately, however, important papers such as those by Alan Turing are readily available on the world-wide web). Furthermore, language itself plays a very important role here, in the way it conveys its message. Some languages and cultures prefer to begin with abstract and general exposition and progress to concrete factual evidence, whereas others are more comfortable if they can first assimilate the data and then move on to the theory. This review has been written with both preferences in mind, and those readers who prefer the concrete to abstract reading order may start reading at The Cell as a Turing Machine, where the ideas are directly linked to experimental data, and then come back to the more abstract paragraphs that begin the review.

## Historical background of the concepts that place *information* at the heart of molecular biology

In its modern form, biology is a recent science. Following the inventory stage, in which species were defined (Daudin, 1926–1927), the first steps in modern biology were mainly concerned with identifying and analysing the lowest relevant level at which those material processes perceived as specific to life could occur. The level of molecules and macromolecules was the obvious candidate (Edsall, 1953): biology had to be analysed in molecular terms in order to move on to prediction, understanding and explanation. Yet, in parallel, the laws of heredity did not rely on molecules in any straightforward way. Genetics was mainly an abstract but rigorous way to account for the laws that directed the transmission of heredity. Molecular biology, which combines the assets of genetics and biochemistry, was born just six decades ago, and has produced most of the concepts on which biological research is now based (Danchin, 2003; Sarkar, 2005).

As in the preceding age of biology, with the concept of species, molecular biology started by building up an inventory of its objects of interest. Its contours had to be outlined, and its ‘atom’, the cell, redefined, along with the various processes that produced that cell. The concept of the ‘genetic program’ began to take on its real meaning in the mid-1960s, when the correspondence between the genes and the proteins, via the rule of the genetic code, was first understood. When DNA sequencing became possible, progress accelerated: in 1982, the sequence of the 50 000 bp of bacteriophage lambda was entirely determined using shotgun sequencing of its randomly fragmented DNA (Sanger *et al.*, 1982). In 1991, it was the turn of a whole chromosome of baker's yeast [300 000 bp (Oliver *et al.*, 1992)] and of a continuous segment of 100 000 bp of the chromosome of a model bacterium, *Bacillus subtilis* (Glaser *et al.*, 1993), which were presented at a European Union meeting in Elounda, Crete. With these sequences, genomics was born, complementing genetics. This was accompanied by a completely unexpected discovery: at least half of the genes found were previously unknown, whether in structure or in function (Danchin, 1995). Subsequently, in 1995 the first complete bacterial genome was deciphered (Smith *et al.*, 1995). Genomics created a new domain in which global rather than local properties of genomes could be studied. Fifteen years later, with the knowledge of the sequence of several hundreds of microbial genomes and a fairly complete picture of the human genome, it was time to see whether we understood what life is. And so began the era of Systems Biology and, more recently, of Synthetic Biology. [While the word ‘system’ is remarkably vague, and ‘synthetic’ emphasizes the role of artifice in the construction of cells, it may be better to stress the role of integration in the new trends of biology. The work ‘symplectic,’ constructed from the Greek, πλɛκτɛιν, to weave, and συν, together, would be more appropriate (de Lorenzo & Danchin, 2008). This is more so because this word has no connotation associated with it that would prevent intrusion of irrational discussions in a purely scientific context.]

Progress in science requires progress in technology. Among the many remarkable features of modern biology is the pervasive need for computers to create and manage biological information. Indeed, it is certainly not by chance that computing and modern biology developed in parallel. This was both for technical reasons (an interesting parallel: 1986, the first GigaFlops machine, 9 million base pairs at the EMBL/GenBank database; 1997, the first TeraFlops machine, 1 billion base pairs at the DDBJ/EMBL-EBI/Genbank database; 2008, the first PetaFlops machine, >200 billion base pairs at the International Nucleotide Sequence Database Collaboration), and, as we shall see, for conceptual reasons as well. As a consequence, alongside *in vivo* and *in vitro* experiments, we now developed a third mode of exploration of life, that of *in silico* experiments (Danchin *et al.*, 1991). This approach is essential not only because of the wealth of data we need to mine and manage, but perhaps – and this is the stance taken in this article – because there is a deep relationship between information and computing on the one hand, and what has usually been taken as a metaphor, the genetic program, on the other. It should be stressed at this point that, while most investigators still only accept experiments as valid when they are performed *in vitro* or *in vivo*, we should shift our notion of proof to include the *in silico* world of demonstration. Indeed, there are conditions under which experiments at the bench result in a disputable outcome, while *in silico* demonstrations may produce unequivocal answers to important biological questions (Iyer *et al.*, 2001). In short, an *in silico* demonstration may occasionally be more appropriate than an *in vivo* or an *in vitro* experiment.

## Concepts common to molecular biology and computer science

A great many articles and books have been devoted to the history of molecular biology and its associated concepts (for a recent avatar, see Manchester, 2008 for instance). However, as one might expect, given that historians of contemporary science necessarily write from an insider's viewpoint, mainstream history often lacks perspective. Hence, it can be difficult, when reading contemporary studies, to spot the trends that will help us to see where the future of molecular biology lies. As in Game Theory (which is deeply connected to the study of evolution), ‘Common Knowledge’ has to be made explicit by outsiders, to permit fruitful inferences to be drawn (Ledwig, 2006). (Common knowledge modifies the action of an agent when it knows that the knowledge it has is shared by other agents.)

Everyone, however, agrees (see e.g. Corbellini, 1998) that the book *What is life*? Written by Erwin Schrödinger at the end of the Second World War had a seminal influence on the creation of the new development in biology that was to become *molecular* biology (Schrödinger, 1945). Not many observed that Schrödinger's insight was in part jeopardized by an ideology of degradation that prevailed between the two World Wars. For example, Schrödinger identified entropy with disorder (still a very popular view, despite the difficulty of defining what order is), and misleading ideas about information and the role of the second law of thermodynamics kept spreading, preventing the development of novel analyses of the future of biology among the other sciences (Danchin, 1986). This unfortunate trend developed despite the important footnotes added by Schrödinger himself, in which he stressed that his physicist colleagues disagreed with his own view of life as a constant fight against the general trend of entropy increase (Schrödinger, 1945).

In parallel, Jacques Monod and many others emphasized the role of chance, a fairly fuzzy concept (see below a mathematical definition of randomness in strings of symbols), as essential to account for a large proportion of the unexpected properties of life (Monod, 1971). This emphasis on chance and noise was, interestingly, based on a misquotation of the pre-Socratic philosophers [very little remains of their words, and so it is fairly easy to check any quotation (Diels, 1902)]. The spurious quotation used as the epigraph of *Chance and Necessity*, and attributed to Democritus, was combined with a profound misunderstanding of pre-Socratic philosophy (Danchin, 1986). Curiously, this emphasis on chance was not challenged by those who knew both the content of the Atomists' thought and the fairly short remnants of their sayings (Diels, 1902). This awkward situation perhaps reflects the unfortunate divide of *The Two Cultures*, which, in many quarters, separates Science from the rest of Knowledge (Snow, 1993). Unfortunately, it had, important consequences, limiting the spread of the understanding of the concept of information, especially in its involvement in biological systems. We shall take some pains to bring the concept back here where it belongs.

Although it did not explicitly acknowledge the fact, Schrödinger's work displaced the emphasis usually placed on the process of *reproduction* as central to life, replacing it with that of *replication* (Dyson, 1985). And as a result, the quest for his ‘aperiodic crystal’ culminated in 1953 with the discovery of the DNA double helix (Watson & Crick, 1953). With the focus now on replication, another shift of perspective occurred: from substrates, biological molecules became *templates* (Danchin, 1983), opening the door for a reflection on information. This shift paved the way for the essential concepts of molecular biology: gene expression and transcription (with the discovery of mRNA), and translation [with the discovery of the genetic code and its (quasi)-universality]. The novel paradigm was summarized in the concept of a genetic program that had no more ‘escaped the notice’ of investigators than did the mechanism of replication when the structure of DNA was discovered (Watson & Crick, 1953), although it was often thought to be merely a metaphor.

The metaphor of the genetic program was a convenient way to describe how cells live and develop. It stated that something stable had to be transmitted from generation to generation, in a way that was more faithful than reproduction would be (it can get away with being fuzzy, provided it is perennial) and was typical of replication (which needs to be as exact as possible). In Schrödinger's view, what had to be transmitted down the generations was not the final organism, but, rather, a recipe to make it (replicating recipes is not difficult to imagine, even though the question of errors during replication must be included in the picture). Replication of a program had the merit of solving the preformationism/epigenesis dilemma, by stating that what is transmitted over generations, when replicated, is the recipe for constructing the organism. For some time, the conceptual success of this solution to a long-standing paradox had disguised the fact that the organism has to be constructed, i.e. reproduced, and not exactly replicated. We shall come back to this point at length below.

In this context, those unusual organisms, the viruses [were they alive or not alive? (Villarreal, 2004)], behaved as autonomous pieces of programs – no virus can survive without a living host cell, using the cell as the machine needed to make the virus multiply and subsequently propagate. In short, they were manifestations of the program, not of the machine that reads the program. Later on, and in a completely different area, when computer programming took off on a large scale, pieces of programs were found to behave in formal terms as biological viruses do, and were named ‘viruses’ accordingly. This was a further indication that the ‘program’ metaphor of heredity was not merely superficial, but perhaps had a deeper meaning.

At least two further concepts were associated with the development of molecular biology. They are central to the engineering view of the cell that prevails in systems and synthetic biology (Kuldell, 2007). The role of *control* (regulation), via feedback (or feedforward and the like) loops (see e.g. Gorini, 1958), as in the lactose operon or in the bacteriophage lambda lytic/lysogenic transition, makes gene expression similar to electronic devices (D'Ari & Thomas, 2003; Alon, 2006). Although it is rather new in biology, the concept of feedback, which has been well understood since the XIX century, is one of the standard concepts of mechanical (‘clockwork’) processes. Much discussion and many experiments have involved feedback and feedforward loops, with their ‘nonlinear’ avatars in particular in systems biology (Alon, 2006; Barrett *et al.*, 2006; Laub *et al.*, 2007; Mitrophanov & Groisman, 2008). Despite its apparent modernity, this domain of biology is therefore typical of the Newtonian world that dominated the XVIII century [see the vogue of automata at that time (Offroy de la Mettrie (translation 1996))].

In sharp contrast, the role of coding in translation, which allows proteins to control protein expression, brought the novel and deep concept of *recursivity* into the heart of biology (Hofstadter, 1979), making cells fundamentally different from mechanical automata in the sense that they are capable of being creative in the strongest sense of the word (Danchin, 2003).

## Life and computation

The discovery of the processes that organize the regulation of gene expression, followed by that of the genetic code, spread the idea that life could be represented as the result of the expression of a program, viewed as a linear string of symbols, the chain of nucleotides in DNA (Liberman, 1979; Yockey, 1992). In a well-known paper a few decades earlier, Turing had proposed that all computations involving integers, as well as all operations of logic, could be performed by a simple machine reading and modifying a tape carrying a linear sequence of symbols, the Universal Turing Machine (Turing, 1936–1937). The concept of the genetic program developed at a time when the first computers had been shown to operate as predicted by Turing, von Neumann and the many theoreticians and scientists who had discovered the link between the arithmetic of whole numbers and logic [Turing, 1946 (1986); von Neumann, 1958].

The most important feature of Turing's model is the requirement for a physical separation between a string of symbols, the *data/program* and a *machine* endowed with specific properties that enable it to manipulate (read and write on) the string of symbols. The genetic program is carried out by the string of nucleotides that make up the DNA molecule. In terms of Turing machines, this raises the straightforward question: can we consider the program to be a *separate* entity in the cell, and if so, to what extent? The basis of genetic engineering is the manipulation of DNA molecules (real or artificially constructed ones) and expression in foreign cells: this is a first proof of concept. Pieces of a genetic program can be transplanted from one organism to another: many bacteria now produce human proteins. Furthermore, not only is it conceivable to construct cells that perform logical tasks, this has been experimentally performed (Elowitz & Leibler, 2000; Buchler *et al.*, 2003). However, these experiments make use of only a small part of the genetic program: can the analogy be extended further, to the whole genome? After the discovery of natural transformation, which identified DNA as the carrier of the genetic program, the discovery of bacterial sexuality suggested that the exchange of a considerable number of genes is widespread in the bacterial world (Hayes, 1952). Later on, the unexpected identification of extensive rather than exceptional horizontal gene transfer in the extant genomes of bacteria (Médigue *et al.*, 1991; Hilario & Gogarten, 1993; Lawrence & Roth, 1996; Baumler, 1997) lent further substance to the separation between the program and the machine, as it was clear that a large number of genes coming from the outside can be expressed and ‘understood’ by any type of bacterium.

**Fig. 1:**
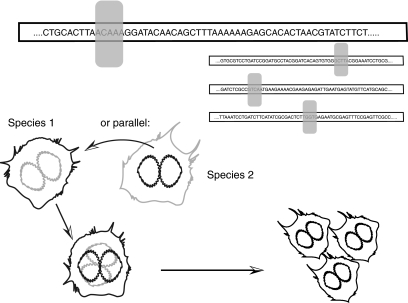
A Turing machine involves physical separation between a machine and the program it expresses.

The considerable importance of this observation, and the fact that it is widespread in newly sequenced genomes (Moszer *et al.*, 1999), did not however, provide final proof that the program defining an organism could be extracted as a whole and placed in another environment, where it could function. In the case of higher eukaryotes, the cloning of the ewe Dolly gave a hint that this might be true (Wilmut *et al.*, 1997). However, a nucleus is not naked DNA, and one could object that, in animal cloning, much of the information was carried by something other than DNA. Proof that the genetic program, carried by a chromosome, was independent, and sufficient to promote the construction of a cell, was finally provided by the recent transplantation of an entire genome from a given species to a different one (Lartigue *et al.*, 2007). This conceptual advance perfected the analogy of the cell as a Turing machine by showing a complete separation between the cell machinery, which will need to reproduce itself, and the data/program, which replicates. Indeed, the latter work proved that a genetic program from one organism could be placed in another organism of a *different* species, and would then propagate as the organism defined by the program, instead of the organism of the initial receiving machine (Fig. 1).

In this context, it becomes even more remarkable that all the processes of molecular biology are algorithmic in their construction. Typically, replication, transcription and translation have the same form: ‘begin – core action – check point – repeat – end’, with the core action being the extension of a polynucleotide or a polypeptide chain. While check points have been studied in the case of replication (Yarmolinsky, 2000), this has rarely been done for the other processes, although there are some examples that suggest a coordinating role for specific codons in translation, for example Thanaraj & Argos (1996). In contrast, standard systems biology follows two different trends. The first aims to represent protein or metabolic networks, and attempts to show that models predict the behaviour of the cell's metabolism (more often than not, this is a retrodiction, i.e. using modelling to find what is already known; for a recent example, see Price & Shmulevich, 2007). The second trend describes the logical networks of regulatory interactions, endeavouring to mimic the logical organization of gene expression (Elowitz & Leibler, 2000; Buchler *et al.*, 2003; D'Ari & Thomas, 2003; Alon, 2006). Hence, it is curious that in general, systems biology does not set its developments in the framework of the algorithmic construction of processes, and, as a consequence, it does not take recursivity into account. Information is not (yet) a central category in this new discipline (de Marco, 2008).

The reluctance of investigators to regard information as an authentic category of Nature suggests that, at this point in the present review of the literature, it may still be difficult for the reader to accept that a cell could behave as a computer. Indeed, what would the role of computation be in the process of evolution? We have already provided some elements of the answer to the question: Turing showed that the consequence of the process of computation along the lines he outlined is that his machine would be able to perform any conceivable operation of logic or computation by reading and writing on a data/program tape. Stated otherwise, and in a way that is easier to relate to biology, the machine manipulates information and, because arithmetic is incomplete [as illustrated in the introduction above (Hofstadter, 1979)], it is able to *create* information. The machine is therefore in essence *unpredictable* (Turing, 1936–1937), but not in a random way – quite the contrary, in a very interesting way, as lack of prediction is not due to lack of determinism, but due to a creative action that results in novel information. If the image is correct, then it shows that living organisms are those material systems that are able to manipulate information so as to produce unexpected solutions that enable them to survive in an unpredictable future (Danchin, 2003, 2008a).

Living organisms are, therefore, infinitely far removed from the clockwork mechanicism that superficial opponents of molecular biology associate with the widespread analytical stance they call ‘reductionism’ (Lewontin, 1993). It is important to emphasize here that, in the Turing machine, the machine is not only allowed to read the program but also to write on it. If, then, the conjecture of the cell as a Turing machine is valid, apparent paradoxes such as the controversial ‘adaptive mutations’ that enable the cell to invent novel metabolic pathways should not be unexpected (Cairns *et al.*, 1988; Danchin, 1988b). We shall discuss this remark further below. At this point, it now becomes essential to explore the concept of information in more depth, in connection with the successor of molecular biology, genomics and its avatar, systems biology.

Finally, we must note that the algorithmic approach, presented when considering the genetic program as an authentic program in a Turing machine (Danchin, 2003), identifies two completely different levels: the level of the program and the level of the machine. This distinction is conceptually essential, and makes it possible to avoid the widespread confusion between replication and reproduction (Danchin, 2008a). This difference, which we will develop further, was vividly demonstrated by Freeman Dyson in his short book about the origin of life, which he deliberately entitled, *Origins of Life* in the plural, to stress the difference between origin of replication and origin of reproduction, the latter being for its most part made up of metabolic processes (Dyson, 1985). Replication, *per se*, results in the error catastrophe pointed out by Leslie Orgel in the case of protein synthesis (Orgel, 1963) and often recognized as Muller's ratchet in the case of heredity (Muller, 1932), while reproduction is not doomed to decay progressively (Dyson, 1985).

## Revisiting information

The work of Claude Shannon provides a first level of understanding of information. He established a theory of communication that was intended to account for the (lack of) fidelity in the transmission of linear sequence of symbols. This theory was not concerned with the meaning of the message, but only with the accuracy of its transmission (Shannon & Weaver, 1949). Curiously, although this view of information is quite appropriate when one considers DNA replication (DNA is replicated whatever the meaning of its sequence – and this is what makes the construction of entirely artificial genes possible), it has long been regarded by many as the only possible view of what information is in genome studies (see Hénaut & Danchin, 1996).

Nevertheless, very early on, some investigators became aware of the importance of the concept of information and of its limitations. In the same year as the structure of DNA was discovered, Henry Quastler, who initially trained as a medical doctor, was perhaps one of the first to realize the importance of information theory and coding in molecular biology (Quastler, 1953). However, like many of his predecessors and successors, he was more interested in the problem of the brain and of consciousness than in what would later be termed the genetic program. The physicist Hubert Yockey (who had participated in the Manhattan project) followed suit, and organized a Symposium on Information Theory in Biology in 1958 (Quastler *et al.*, 1958). The meeting was quite successful, but rapidly forgotten by biologists, curiously at a time when the reflection on information kept developing in other quarters. In a work published posthumously, Quastler further developed a theory of biological organization, starting with the enigma of the origin of life. The interesting point in his short essay is the emphasis he places on the problem of *creation* of information in simple cells, a question of central importance, as we have already seen (Quastler, 1964; Danchin, 2008a).

The first of the new developments that explored extensions of information theory were the parallel studies of Kolmogorov in Soviet Union, and Solomonoff and Chaitin in the United States in the mid-1970s, which set out to identify the nature of information in sequences of symbols. One goal of these studies was to attempt to provide a definition of a random sequence, which was not a trivial task (see Cover & Thomas, 1991). The concept of *algorithmic complexity* defines a sequence by the shortest algorithm needed to generate that sequence. With this definition of sequence compression, a random sequence will be said to have high algorithmic complexity (it cannot be compressed to a length shorter than itself) while a repeated sequence would be of low complexity. (This definition, which is very precise as are all mathematical objects, illustrates in an illuminating way the ambiguity of the use of the word ‘complexity’ by laypersons. As can be seen, both extremes of algorithmic complexity look ‘uninteresting’. Furthermore, while ‘complex’ in the mass media is used with considerable positive connotations, it is seen here that its highest level is simply equivalent to randomness!) A further development came with the definition of *logical depth* by Bennett (1988a). This latter concept, which is not yet commonly considered when information is discussed, is based on the observation that two sequences with the same algorithmic complexity might differ widely in the way they carry information. For instance, in a repeated sequence, which looks fairly trivial, it is a reasonably straightforward task to find out what any given symbol must be – in other words, to obtain the information of that symbol. In contrast, for sequences produced by a recursive algorithm, it is often impossible to infer the nature of the symbol without running the algorithm, and when this symbol is located far downstream in the digits of the sequence, this can take a very long time (or may be impossible, in any predictable future). The time required to access the corresponding information measures the logical depth of the sequence.

As an example of the interesting and nontrivial features of the latter kind – algorithmically simple but logically deep algorithms – consider fractal figures such as Koch's snowflake or the Mandelbrot set. Both are generated by fairly short algorithms, but the outcome of the algorithm cannot be predicted easily before it is run. We will not go further on this point in this review, except to note that it is an important point to consider when analysing phylogenies. Indeed, because DNA makes DNA, makes DNA … through generations, it must be admitted that any nucleotide may have a considerable logical depth. This strongly suggests that there is no such thing as really ‘junk’ DNA (Danchin, 2003). Finally, as a support to our interest in the concept, a further essential role of information is now seen in physics, where it is considered to bridge the gap between classical physics and quantum physics, solving most of the paradoxes raised by Einstein and his colleagues (Steane, 1998).

Further extending the reflection about the very nature of information may be highly relevant to the processes that involve accumulation of biological information. Although it cannot be discussed further here, as this would take us a long way from microbiology, this type of investigation into the role and form of information in molecular biology is under constant development (Yockey, 1992; Danchin, 1996, 2008a; Liberman & Minina, 1996; Lifson, 2005; Chaitin, 2007). Some of the applications of nonstandard definitions of information in genomics were reviewed a few years ago in the American Society of Microbiology's ‘bible’ of *Escherichia coli* and *Salmonella typhimurium* molecular genetics (Hénaut & Danchin, 1996). As an example, the concept of sequence complexity, which was widely used to define different classes of DNA by hybridization before the advent of DNA sequencing, is now familiar to all investigators using blast filters (see e.g. Huynen *et al.*, 1998).

As early as 1972, Carl Woese attempted to associate the downstream process of translation with the tape-reading metaphor of the Turing Machine, linking it with the creation of complexity during evolution (Woese, 1972). Later on, opponents of the idea that identifying the molecular level as very important would lead to progress in biology attempted to evoke a chicken and egg paradox in the repeated observation that living organisms create progressively more complex structures and processes (see e.g. Nagel, 1998; Waliszewski *et al.*, 1998). In contrast, several investigators concluded that cells could be regarded as authentic information-managing systems, where complexity – provided it is carefully defined – has its place (Danchin, 1983, 1988a, 1995, 1996; Savageau, 1991; Yockey, 1992; Danchin & Hénaut, 1997; Danchin *et al.*, 2000; Maynard-Smith, 2000). However, the idea was generally not well received, mainly because of the profound ambiguity in the term complexity (common knowledge modifies the action of an agent when it knows that the knowledge it has is shared by other agents), which allowed critics to play with words (Danchin, 2003). Once again, several investigators saw molecular biology as ‘reductionist’, without understanding that the analytical method does not reduce a system to its parts (Lewontin, 1993). At present, indeed, a major reason for the widespread (and philosophically ambiguous) interest in systems biology is its integrative (‘holistic’) role: yet molecular biology has already defined the lowest level required for analysis of biological systems, and it is now time to move on to reconstruction.

Generally speaking, because of emotional preconceptions based on long-held, traditional views of the position of Man in the Universe, there has been a great deal of reluctance to accept that life might be understandable (this does not, by any means, mean predictable!), While this was perhaps permissible when investigators proposed a purely mechanistic view of the cell, as if it were a complicated but standard automaton of the mechanical type, this is certainly no longer relevant in the case of Turing machines, as, once again, cells are constructed in such a way as to be both innovative and unpredictable (Danchin, 1996). The work on information has shown that contrary to intuition, physics does not preclude but permits the creation of information (Landauer, 1961; Bennett, 1988b), so that if the conjecture that cells can be seen as Turing machines holds, then their ability to create new forms and processes is fully in accordance with the laws of physics (Danchin, 2003, 2008a).

## The cell as a Turing machine

A Turing machine is an abstract entity. In concrete physical terms, it has been implemented in the form of computers. Many constraints are involved in this transition from the world of abstraction to the material world. In particular, the interaction between the machine and the program needs to be made explicit. To make this bridge, von Neumann proposed the concept of what we now refer to as the operating system (OS), a particular piece of the program essential to run the machine (von Neumann, 1958).

### OSs

The guiding principle of the OS is that it links the concrete world to the abstract world of symbols – in our view of information as an authentic category of Nature, it connects information with matter, energy, space and time – by constructing a representation of all the essential relationships in the structures and processes involved in the Turing machine. Within the data/program, the OS defines functions intended to create an image of the processes necessary for the machine to work. The program must first be able to separate between the machine and its ‘users’. Users here are usually not human users, but other machines (printers, screens, memory storage devices and all kinds of peripherals) and some are even programs. This involves implementing a ‘virtual machine’ within the program, which serves to hide from the users all the engineering details of the computer as a physical entity. The OS should also code for a ‘resource manager’ to share the necessary physical and abstract routines efficiently and effectively among users of the machine (each one using and creating data while running programs). In addition to the OS, and in relation with it, several classes of programs must be defined, such as systems programs (loaders, compilers and editors), applications support programs (database management systems and networking systems) and finally, the programs that correspond to the goals of the machine, applications programs. Finally, in the cell as a computer paradigm, because the OS of the cell needs to manage many nanomachines, it is rather expected to be of the object-oriented type (i.e. managing resources inside data files).

Let us note here that, as cells have not usually been viewed with this Turing machine model in mind, the functional categories that have been created to describe biological functions have not been organized in this way. The level of transcription appears to be particularly well suited to fulfil many of the expected functions of the OS [and management of resources can easily be perceived in the nucleotide content of RNAs, for example, linking metabolism to genes (Cohen, 1960; Rocha & Danchin, 2002)]. However, it would probably be rewarding to entirely revamp the so-called ‘ontologies’ (The word ‘ontology’, which has a very specific meaning in philosophy, has curiously been diverted from its original meaning in health care sciences (Herbert, 1995). To refer to a particular vocabulary describing knowledge associated with a patchwork of biological data, objects, sequences, biological functions and functionalities and other general features of biological processes. It then spread to genomics.), which describe biological objects and processes along these lines, and the ‘computer’ view of the cell might be useful in designing a new, structured vocabulary to account for biological structures, functions and relationships.

An ontology aims to provide precise definitions of the objects and relationships in a given domain of knowledge (Herbert, 1995). The main problem faced by the endeavour to create a particular ontology was identified long ago, in a remarkably prescient way, by Myhill (1952). A mathematician and an epistemologist, Myhill analysed the way logic uses what he termed ‘characters’ (concepts). ‘Effective’ characters can be immediately transmitted from one person to another one, without ambiguity. ‘Constructive’ characters need some thought on the part of the receiving person, and then understanding is common to emitter and receptor: this is the result of a straightforward logical computation. Prospective characters are understood in a way that changes every time they are discussed: they derive from recursive computation and as a consequence their meaning is altered during each exchange. Many concepts in biology, and in particular the concept of ‘function’, are prospective, so that they do not fit comfortably under the yoke of an ontology. Frequently associated with the idea of ‘function’, the concepts in an ontology are very fuzzy, and are not used consistently by biologists (Allen *et al.*, 1998). The consequence is that except in narrow domains of intermediary metabolism, the association of an ontological term with a biological object is restrictive, and ill suited to encourage discoveries. Furthermore, because biological objects are often involved at many levels, with different degrees of integration (‘granularity’), it may be necessary to use several ontologies simultaneously or ontologies that combine different levels of integration.

This question is a very important one, which will need further reflection, as the definition of the exact meaning of a particular vocabulary to describe features of genomic objects is an essential prerequisite for genome annotation. Several ontologies are used in this respect, in particular, the GO ontology (Gene-ontology-consortium, 2001, 2008). This classification, although not originally defined for bacterial genome annotation, is useful when considering individual proteins in the context of the cell: what they do, i.e. the molecular function that describes the biochemical role of the protein (transporter, regulator, enzyme, structural protein, etc.); where they are found in the cell, i.e. their subcellular localization (cytoplasm, periplasm, cytoplasmic membrane, etc.); and what larger processes they participate in, i.e. the biological function that describes the role of the protein in the cell (metabolic pathway, signalling cascade, etc.).

### Multiple OSs: the three domains of life, or more?

When these abstract concepts are translated into real lines of code, there is nothing to say that only one type of OS should exist. Indeed, in the computer industry, many exist. OSs are not even fixed in time (remember CP/M-86® and MS-DOS®), and they evolve, as witnessed in today's computers. What do we find in genomes if we keep the Turing machine model in mind? Many articles identify ‘housekeeping genes’ (1226, fall-2008), showing that there is some consensus on the nature of the processes that have to be present in all cells. Cells display highly conserved features, such as the (almost) universal rule of the genetic code, as well as the DNA replication machineries. However, conservation of function is certainly not conservation of structure. For example, cell division is remarkably different between the eukaryotes and the prokaryotes. Compartmentalization is also very different in these organisms, with the former having a well-formed nucleus. In the class of prokaryotes, Woese upset the biological community with his discovery of remarkable discrepancies between two classes of cells: the Archaea and the (previously recognized) Bacteria (Woese *et al.*, 1978). He found that they were distinguished by the very core of their housekeeping machinery (translation first, but also transcription, replication and compartmentalization), and we can see today that even Bacteria are not homogeneous [see the debate about the origin and nature of prokaryotes (Gupta, 1998, 2000; Mayr, 1998; Cavalier-Smith, 2002, 2006)].

This exploration of the OS model provides us with the first level of diversity in prokaryotic genomes, located at a very deep level, and probably originating very early on in the evolution of life: despite some similarities, there are major differences in the housekeeping genes coding for replication, transcription and translation, even within the Bacteria domain. In this context, the experiments of Venter and coworkers in *Mycoplasma* (Lartigue *et al.*, 2007) need to be placed in perspective. Just as we cannot expect that a program meant to run on a MS-DOS® platform will run smoothly on a Windows NT® platform, we cannot expect that the transplantation of any genome into any other cell will be productive. And indeed, when a whole cyanobacterial genome was transplanted into *B. subtilis*, the *Bacillus* did not express the Cyanobacteria genome (Itaya *et al.*, 2005).

The reasons for this can be stated explicitly in Bacteria: for example, there are at least two classes of core DNA polymerase III in these organisms. Most use only one DNA polymerase to manage both DNA strands, while the A+T-rich *Firmicutes* use two such enzymes (DnaE and PolC), perhaps for a different management of the leading and lagging strands (Rocha, 2002). Symmetrically, the *Firmicutes* use only one SpoT/RelA protein both for synthesis and for degradation of the universal regulator pppGpp, while *Gammaproteobacteria* have two such enzymes: SpoT and RelA (Hogg *et al.*, 2004).

As another example, RNA metabolism differs in different bacterial clades, retaining the same functions, but not the same structure, with a degradosome that is widely different in *Gammaproteobacteria* and in *Firmicutes* (Danchin, 2008b). In summary, there is an in-built diversity that fits not only with the three domains of life but with smaller clades as well. This implies that in systems biology approaches, one should not extrapolate too early from a particular organism to another one. If we hope to be able to understand the highly parallel organization of gene expression, novel approaches will have to be implemented to deal with the large number of features associated with the many relationships built up within cells. This will require a general effort aiming at a ‘two-dimensional’ annotation of genomes (Palsson, 2004).

At this point, we can reconsider the common reluctance to see the cell as a computer. The usual objection raised is that the cell's information content is much higher than that of its chromosome. With the points discussed above, this objection does not hold. Or, rather, one could raise exactly the same point with authentic computers, which nobody would deny are material implementations of Turing Machines. The concrete machine that enacts a program does comprise much more information than is in the program it runs. A further negative objection is that, in a cell, it is not possible to completely separate the hardware from the software. However, this too is exactly mirrored by the situation of the program coding an OS. While an OS is an abstract entity, to be usable, it must be carried by concrete objects, such as a compact disk (CD). A CD left lying for some time in a car's rear window in the sun will be deformed, and despite the fact that the program it carries is unaltered, it will no longer be read by the computer's laser beam, and so the computer cannot use it to start up. In other words, although in the abstract world in which Turing Machines exist the separation between hardware and software is rigorous, in practice, there must be a physical support for each entity, and so we cannot completely separate the hardware from the software in any real implementation of the Turing Machine. This is an important constraint that may create difficulties in transplantation experiments such as those where an artificial *Mycoplasma* genome has been synthesized, using *Saccharomyces cerevisiae* as an intermediary host (Gibson *et al.*, 2008): it could well be that the resulting folding of the chromosome makes it unreadable by the receiving *Mycoplasma* machinery. Indeed, at the time of this review article, no transplantation experiment has yet been published using this synthetic construct (Peter *et al.*, 2004; Peckham *et al.*, 2007).

Further refinements can also be identified in the OS model. Bacteria are not always single-cell organisms. Sometimes, as with several Cyanobacteria or with Streptomycetes or Myxobacteria, they are multicellular. In single cells, one expects an OS similar to that of personal computer OSs, with some time-sharing properties. For more complex organisms, distributed systems would obviously be needed. All this demonstrates that in investigating essential functions, we should proceed with caution: once again, while the functions need to be conserved (and some of them might be specific to particular states of the organisms, with multicellular organisms differing from unicellular ones), there is no compelling reason why these genes should have to have exact sequence counterparts in all organisms. The only good reason for universality would be historical: if it is difficult to create this or that function, it is likely that once it has appeared somewhere it will spread everywhere. This implies divergent evolution (but horizontal transfer as well). In contrast, for functions that are more straightforward to create, it could be a case of convergent evolution.

## Global rules of genome organization

At this point in our reflection, we have seen that the cell, the atom of life, can be considered as a machine manipulating the information carried by a program. We have been led to consider that the machine and the program are separated, as they must be in a computer, a Turing Machine. Of course, computers do not make computers. Very simple automata such as crystals can reproduce, but as soon as they are at all complicated, this apparently becomes impossible. If we had to think of a computer that makes a computer, what would be the constraints be? In a paper based primarily on the insight of a deep [not straightforward (Trautteur & Tamburrini, 2007)] analogy between the brain and the computer, von Neumann proposed that, within the computer, there should be some kind of image of the machine, which would also be passed on from generation to generation (von Neumann, 1958).

While in the world of abstraction the program and the machine must be separated, in the concrete world they need to be somehow linked together. In living organisms, the most obvious hereditary component is the chromosome, and so it is interesting to explore whether, and how, some image of the cell could be built into the way the chromosome is organized. In order to do so, we first analyse the literature dealing with the way DNA is handled by the various machineries in bacteria, explore the diversity of the corresponding processes and then try to see whether, despite this diversity, some common features emerge.

### Physico-chemical constraints on the bacterial chromosome

To explore the organization of the bacterial genome, we must identify the various constraints to which the genome is subjected. As a long, partially rigid polymer, DNA has to fold into a tiny space. In the presence of the physiological concentration of ions, its persistence length (average rigidity) is of the order of 50 nm (150 bp) (Kebbekus *et al.*, 1995). In *E. coli*, for example, if the DNA were randomly folded it would occupy a sphere with a diameter 10 times that of the normal cell. This shows that superordered DNA structures need to be considered to account for its packaging in the cell. A wealth of studies have explored the variety of constraints that operate on DNA: supercoiling, domain structure and attachment to specific sites (Haran *et al.*, 1994; Pedersen *et al.*, 2000; Tolstorukov *et al.*, 2005; Zimmerman, 2006). There are some indications that these physical constraints are reflected in the genome sequence in the form of fuzzy motifs (named ‘flexible motifs of type A’) that constrain a considerable amount of the DNA sequence (Larsabal & Danchin, 2005).

Packaging DNA into a tight volume strongly limits the space and energy states available to the molecule. This means that when the size of the compartment grows, the degrees of freedom available to DNA increase. As a consequence, there is a spontaneous entropy-driven tendency of a replicating DNA molecule to occupy the space offered by cell growth (Brochard-Wyart *et al.*, 2005), creating a natural process for DNA segregation (Danchin *et al.*, 2000). Indeed, explicit modelling of a situation in which two long polymer molecules are mixed in a small chamber, under conditions similar to those of replication, shows that an entropy-driven process will tend to segregate the molecules, in precisely the opposite direction to the standard mixing of Boltzman's gases (Jun & Mulder, 2006).

### Constraints imposed by replication and transcription

Replication has to start either at a fixed origin, or more or less randomly along the chromosome. Because DNA is made of two strands oriented in opposite directions, a topological problem is posed at the extremities of the molecule, in a linear chromosome or at the knotted structure formed when replication terminates, in a circular chromosome. In the former case, the cell needs a specific process to manage telomeres to take care of the necessary overhangs required for attachment of the DNA polymerase replicating the lagging strand (Bankhead *et al.*, 2006; Jayaram, 2007), whereas in the latter situation, special enzymes must cope with an accumulation of superhelical turns and cleavage of the knotted structure formed at the terminus (Corre & Louarn, 2005). For this reason, if an origin exists, there is usually a particular distribution of genes around it (Horimoto *et al.*, 2001; Takeuchi *et al.*, 2005; Maeder *et al.*, 2006) and around the terminus as well (Horimoto *et al.*, 2001; Lindroos *et al.*, 2006; Berger *et al.*, 2007).

The biochemical processes and the physics of replication are entirely different for the leading and lagging DNA strands (Fijalkowska *et al.*, 1998). This results in considerable bias in all features of the DNA sequence, with important consequences for gene and protein composition (Lobry, 1996; Rocha *et al.*, 1999; Rocha & Danchin, 2001; Lobry & Louarn, 2003). The dissymmetry in the organization of the chromosome has an enormous impact on gene organization, as it opens the door for conflicts between transcription and replication. Replication is much faster than transcription (French, 1992). If both processes occur along the same strand at the same time, the solution of the conflict is simply that replication slackens its pace when it meets active transcription (Wang *et al.*, 2007). However, when transcription and replication meet head-on, this results in a series of deleterious outcomes (Mirkin & Mirkin, 2005). While these conflicts are solved at the level of DNA itself (Rudolph *et al.*, 2007), the formation of a truncated mRNA remains extremely damaging, so much so that evolution has invented a rescue system involving a special RNA, tmRNA, to cope with truncated mRNAs and the corresponding truncated polypeptides they generate (Haebel *et al.*, 2004).

The consequence of these constraints is that in general the distribution of genes along the leading and lagging strands of the chromosome is uneven, with a particularly large bias in *Firmicutes*, where many more genes are located in the leading strand than in the lagging strand. The avoidance of formation of truncated proteins is further reflected in genes that are essential for life: they are almost always located in the leading strand of bacterial genomes, whatever their level of expression (Rocha & Danchin, 2003a, b).

### Translation organizes the genome

Three decades ago, Grantham proposed multivariate analysis of codon usage bias as a means of identifying specific genome signatures (Grantham *et al.*, 1980). While the first studies revealed the existence of two major classes of genes (Gouy & Gautier, 1982), Médigue *et al.* (1991) made the unexpected discovery that in *E. coli*, horizontal gene transfer involved a considerable number of genes, and that this involved a characteristic feature of the corresponding codon usage bias. Further work expanded this observation, and suggested that the biochemical process of translation *in vivo* had a considerable impact on the way the genetic code was used, suggesting a link between the process of translation, the architecture of the cell and the organization of the chromosome (Danchin & Hénaut, 1997; Guerdoux-Jamet *et al.*, 1997; Nitschké*et al.*, 1998; Danchin *et al.*, 2000). Multivariate analyses showed that functionally related genes had related codon usage biases (Nitschké*et al.*, 1998; Fuglsang, 2003), but this was not explicitly related to the genome organization.

A recent study introduced information as a central element in the analysis. A novel approach based on assigning all coding sequences in a genome to *N* clusters, while looking for the best partition in terms of information content, revealed that the codon usage distribution along the chromosome was far from random (Bailly-Bechet *et al.*, 2006). This work showed that a specific role of the diffusion of some tRNA species is a likely cause of the nonuniform nature of genome organization. This suggests that many of the models used in systems biology rely on hypotheses (continuous differential equations in particular) that are often too crude to offer a realistic representation of the cell. There is some indication that this translation-driven organization is also visible in global transcription patterns: in *E. coli*, transcription patterns could be classified into three categories: short range, of up to 16 kb; medium range, over 100–125 kb; and long range, over 600–800 kb (Jeong *et al.*, 2004).

## Functional rules of organization

Models have been proposed to account for these organizational constraints and their relationships with various aspects of the cell's architecture (Takeyasu *et al.*, 2004; Luijsterburg *et al.*, 2006; Woldringh & Nanninga, 2006). However, while long-range effects demonstrate that genes may be far apart in the genome, but neighbours when the chromosome is folded up in the cell, no experimentally validated model of organizational rules has yet emerged (Esnault *et al.*, 2007). In particular, despite considerable constraints on folding, no regular overall structure of the bacterial chromosome has yet been identified. The question then arises as to whether specific biological functions influence the way genes are distributed in the genome.

### Metabolic clusters

In addition to processes related to gene expression, there are also constraints driven by metabolic features. Bacteria that multiply very fast tend to use the gene multicopy effect around the origin of replication to favour there the presence of genes that need to be expressed at a high level under exponential growth conditions (Couturier & Rocha, 2006). Genes involved in processes that need to be compartmentalized because they involve highly reactive intermediates, such as sulphur metabolism, form clusters. Sulphur metabolism genes, for example, are grouped into islands in *E. coli* (Rocha *et al.*, 2000), and the situation is quite similar in *B. subtilis* (Sekowska *et al.*, 2000). In the same way, transport and degradation of carbohydrates often form clusters of genes, with related functions, but not always related structures (Plantinga *et al.*, 2004). If these constraints are efficient, then it is likely that comparing many strains of a given species will show conservation of a backbone of genes, with little disruption by invading horizontally transferred genes. Early observations with the *E. coli* genome support this (Brzuszkiewicz *et al.*, 2006).

### From gene persistence to genome organization

In exploring the principles that organize genomes, some investigators have conjectured that complexes sometimes named ‘hyperstructures’ are formed within the cell. They are thought to be responsible for the shape of the sacculus (Egelman, 2003; Errington, 2003; den Blaauwen *et al.*, 2008) and to constrain the distribution of genes in the chromosome (Rocha *et al.*, 2003). Furthermore, various experiments have shown that the bacterial cytoplasm is far from being a tiny test tube, but a structure that is quite firmly organized by the chromosome and by other complex structures (Lewis & Errington, 1997; Sharpe & Errington, 1998; Webb *et al.*, 1998; Ben-Yehuda *et al.*, 2003). These observations derive from *in vivo* experiments, which cannot be easily duplicated in many organisms, and it is difficult to know how general they are. *In silico* analyses are therefore well suited to tackling the question of the underlying organization of the cell.

Before proceeding in our quest for rules or organization, it is essential to make explicit a constraint that drives all living systems and makes comparative genome analyses difficult. Briefly, the triplet that drives evolution, variation/selection/amplification, constantly opens up niches for invention of new functions. However, these functions can only be performed by objects that must either be recruited from previously existing objects or created *de novo* (Danchin, 1989, 2003; Allen *et al.*, 1998). The consequence is that there is no one-to-one relationship between the structure and the function of a biological object (Danchin, 1999). Even if essential functions need to be preserved in all genomes, this never implies that the corresponding structures have to be the same. Comparative phylogenetic analyses will only provide us with a partial view of the functions we are interested in.

Strictly speaking, when we attempt to identify those functions that are ubiquitous, we are limited to the study of the structures (and even worse, of the sequences) that are present in some reasonably chosen fraction of all the genome sequences available. Fortunately, we have a way out: living organisms form a chain of descent, so that there is a tendency in the lineage to stick to one object when that object fulfils a given function. Hence, within a particular group of organisms, it is most likely that the structure/function relationship will often hold. From time to time, a discontinuity will be observed, corresponding to the moment when a particular object is replaced by a new one. With these constraints in mind, it is efficient to look for gene ‘persistence’ in genomes (i.e. look for genes that are present in a number of genomes, but not necessarily in all genomes) and to further study the way persistent genes behave functionally and in the course of evolution (Fang *et al.*, 2008). With several hundred genomes available, it became possible to study *in silico* not only the presence of persistent genes in genomes (Fang *et al.*, 2005) but also to analyse the way their relationships are conserved.

### The paleome, the cenome and the minimal genome

In the early days of genome projects, it was thought that knowing many genomes would make it possible to identify a genome with the lowest possible number of genes compatible with life: a minimal genome. This goal was indeed proposed to justify applications for support from research agencies for genome projects (Danchin, 1988a). When the small genome of *Mycoplasma genitalium* was deciphered, it was used as a blueprint to identify the genes that would make up the minimal genome, implicitly assuming that sequences (structures) formed a one-to-one correspondence with the functions essential for life (Mushegian & Koonin, 1996). However, as the number of known genome sequences increased, the set of ubiquitously conserved genes kept decreasing (Carbone, 2006). It now appears that, rather than using the intersect of conserved genes in all genomes as the basis for the minimal genome, it is necessary to start from a consistent gene set present in a given species, progressively trying to reduce it, while keeping the cell alive.

**Fig. 2:**
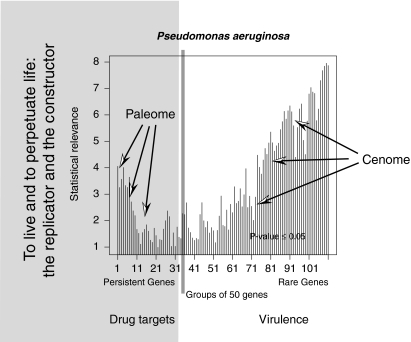
The paleome and the cenome [adapted from Supplementary Figure 1, p. 76 Fang *et al. Proteomics* (2007) **7:** 875–889]. Grouping genes according to their frequency in bacterial genomes (groups of 50 genes), with increased rareness (common genes on the left and rare genes on the right) reveals that both frequent genes and rare genes tend to remain clustered together in genomes (the horizontal lines gives the limit for statistical significance of grouping). Four hundred to 500 frequent genes (persistent genes) tend to stay clustered together despite the frequent shuffling and horizontal gene transfer in genomes.

The way to tackle this question is to start from the set of persistent genes and study the way they are organized in genomes. Analysis of conservation of syntenies in genomes showed that both persistent genes and rare genes tend to remain clustered together (Danchin *et al.*, 2007). Overall, the genes in genomes make two highly consistent families, separated by a large twilight zone that corresponds to genes essential when the cell's nutrient supply diversity is poor (Fig. 2). The first family is made of *c*. 500 genes, which both tend to persist in genomes and to persist in the way they cluster in genomes (Fang *et al.*, 2008). Further statistical analysis demonstrated that persistent genes remain clustered as a network that strongly suggests a mineral scenario of the origin of life (Danchin, 1989). This set has accordingly been named the *paleome*. (From παλαιος, ancient; cenome is from κοινος, common, as in biocenosis, and instead of coenome, which would be more correct, but with a rather awkward spelling; c.f. oecology vs. ecology.) Briefly, the genes of the paleome form three sets, which differ in terms of the way their connectivity is preserved during evolution. A first set, in which clustering is poorly conserved in genomes, codes for synthesis of the basic building blocks that cells are made of: amino acids, nucleotides, coenzymes and lipids. A second set is organized by connection to class I tRNA synthetases, and it also comprises genes permitting cell division. The third set, highly connected, is organized around the machineries of transcription and translation, with the ribosome as its core structure (Danchin *et al.*, 2007). The functions of many of these persistent genes are understandable: they contribute to the construction of the cell and to replication of its genome. However, a considerable proportion of them are involved in functions that appear to be related to maintenance and repair (Fang *et al.*, 2005). Furthermore, this latter class is not strictly essential, as the corresponding genes can be inactivated without total loss of viability. These genes appear therefore to contribute to the perpetuation of life, rather than to permit life *per se* (Danchin, 2008a).

In contrast, the set of genes acquired by horizontal gene transfer corresponds to genes needed by the cell to survive in a particular environmental niche, not to provide the basic functions for life. This class is very large, and does not seem to be limited in number, as it tends to comprise new members in different strains of the same species. It has accordingly been named the *cenome*, to refer to its role in permitting the organism to live in a particular niche (biocenosis is a common concept in ecology, created by Karl Möbius in 1877, see e.g. Movila *et al.*, 2006; Danchin, 2007).

Some effort has been devoted to constructing minimal genomes, starting from wild-type organisms. This is the goal of work by Claire Fraser, Hamilton Smith, Craig Venter and colleagues, on the genome of the already highly compact organism *M. genitalium* (Hutchison *et al.*, 1999). Because this organism has such a small genome, studying it cannot provide much information in terms of clustering rules, as genes are necessarily close to each other. By contrast, reduction of the *E. coli* (Posfai *et al.*, 2006; Mizoguchi *et al.*, 2007) or the *B. subtilis* genomes (Ara *et al.*, 2007) is much more rewarding in this respect. While we are still a long way from very compact genomes, it is interesting to observe that the fitness of the organisms under laboratory conditions does not appear to have decreased in parallel with the first attempts in genome reduction, but perhaps even increased. Furthermore, comparison of different strains of the same species tends to show that there is a fairly invariable backbone in the genome, with specific places where foreign genes can be introduced more or less at will (Burrus & Waldor, 2004). This is consistent with the paleome/cenome split in genome organization. Most cenome genes are not essential for life but, rather, enable a cell to cope with the diversity of the situations in a specific niche, at the cost of some fitness for life under very stable and reproducible conditions.

### Is there a ‘celluloculus’?

All these observations show that the order of the genes in the bacterial chromosome is not random, and that there are many sources of selection pressure to organize them together. However, is this linked to a map of the cell? At this point, the reader might still have some difficulty in accepting this conjecture as valid. How would a linear sequence of symbols be connected to an architecture? Chemists, with their Simplified Molecular Input Line Entry System (SMILES) representation of chemicals, provide us with a concrete illustration (Karwath & De Raedt, 2006). How do they represent l-glycerate, and tell it from d-glycerate? The SMILES nomenclature is clear and shows that an architecture can be described by a sequence of symbols belonging to a finite alphabet. Sequences of the same 38 symbols, in a different order, describe each of these molecules: 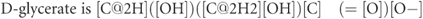

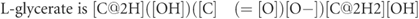


Even better hints for a possible answer may come from the study of multicellular organisms. In the early 1960s, extraordinary mutations were discovered in the drosophila fly: modifying particular genes termed homeotic genes produced mutants that had legs where their antennae should be (Lewis *et al.*, 1980). Many similar genes were discovered later on, including in plants (for reviews, see, Adam *et al.*, 2007; Handrigan & Wassersug, 2007; Iimura & Pourquie, 2007; Schwartz & Pirrotta, 2007). Quite remarkably, the order of the genes along the chromosome seems to match the order of features along the antero-posterior axis of the animal. While this is observed both in vertebrates and in invertebrates, there is no explanation for this remarkable fact, despite the identification of coregulated territories in the cell's nucleus (Heard & Bickmore, 2007). Knocking out a homeotic gene often results in a segment being transformed into a more anterior type of segment. In general, it can be concluded that insects have one such set of homeotic genes, while mammals have four (Bachiller *et al.*, 1994). Finally, strange animals such as the Platypus have a mosaic genome that parallels this animal's fascinating combination of reptilian and mammalian characters (Warren *et al.*, 2008).

We would like to point out here that this is exactly what von Neuman's conjecture would lead us to expect. There is, as yet, no convincing explanation to account for the selective forces that maintained this order in these control genes, making exploration of the conjecture even more interesting. In short, there is an ‘animalculus’ in animals, similar to the ‘homunculus’ that preformists thought they saw at the origin of the development of Man (Danchin, 2003). This novel algorithmic view combines both the pure preformist and the pure epigenetic views of development: an algorithm is not a minute animal, but a physical organization of the program that makes the animal, and to be put into action it needs external inputs, typical of what is expected from the epigenetic view. In any event, there needs to be a rigorous separation between genetic and epigenetic heredity (Danchin, 2003). We note here that this separation also implies a conceptual difference in the underlying processes of duplication: replication for the program and reproduction for the organism.

**Fig. 3:**
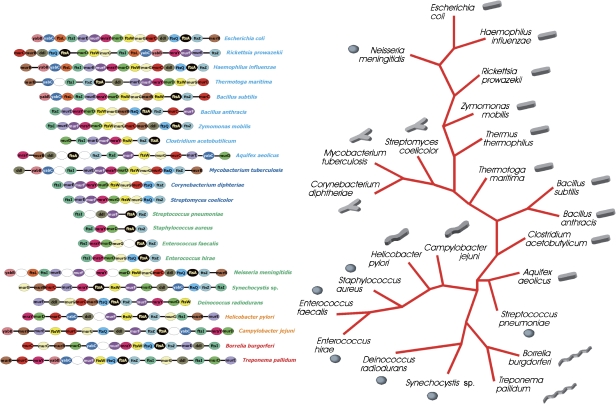
The tree of the distribution of genes in the *mur-fts* clusters does not follow 16S rRNA gene phylogeny, but is consistent with a tree based on the bacterial shape (modified from Tamames *et al.*, 2001). On the left of the figure the *mur-fts* clusters are represented for different organisms. Black bars indicate genes located apart in the genome. Empty ovals represent intervening genes. The name of each species is coloured according to the shape of the cell; blue, bacilli; dark blue, *Actinomycetes*; green, cocci; orange, helicoïdal *Deltaproteobacteria*; red, *Spirochetes*.

Can we point to similar properties at the level of individual cells, bacteria in particular? Is there a ‘celluloculus’? Tamames and coworkers made the bold hypothesis that the conjecture might hold for genes that must be somehow involved in shaping the cell. The organization of *mur-fts* clusters, present in bacteria with a cell wall, is quite variable. These authors uncovered an unexpected pattern of relationships between the order of the genes in the clusters and the shape of the bacteria (Tamames *et al.*, 2001). Most remarkable was the observation that although the corresponding tree fitted both the gene order and the shape of the cell, it did not follow the phylogenetic tree. This finding suggests that the relationship between the order of these genes and the architecture of the cell is a deep one (Fig. 3). This work was further developed, and the authors proposed a model in which the selective pressure to maintain the division and cell wall gene clusters arises from the need to coordinate efficiently the processes of elongation and septation in rod-shaped bacteria (Mingorance *et al.*, 2004). Physical principles are needed to account for this type of organization. While the asymmetry of the cell's volume in Bacilli is perfect to accommodate entropy-driven chromosomal segregation (Danchin *et al.*, 2000; Jun & Mulder, 2006), it would be interesting to explore the organization and expression of the corresponding genes in cocci in depth, as symmetry breaking will be needed to permit the unambiguous splitting of chromosomes into daughter cells (Harold, 2007). In the Archaea, there are even square cells, and it will be interesting to understand the articulation between the information in the genome and this exceptional morphological feature (Walsby, 2005).

## Causes of organization

At this point, we may accept that rules for the organization of genes in the genome do exist, and that the distribution of some genes is correlated with the shape of the cell. A major property of these rules is the explicit tendency of genes to cluster together. Where it exists, clustering is continually counteracted by processes that tend to disrupt clusters. Bacterial genomes tend to exchange genes with others in their environment, constantly gaining and losing genes with a corresponding alteration of their sequence. Another common process of sequence alteration is the very frequent one of local duplication of genome sequences of variable length (Cole & Guest, 1979; Danchin and Ullmann, unpublished observations), which, combined with recombination and mutation, can rapidly make the genome sequence evolve. This process explains why, while gene order is preserved extensively in closely related species, it fades away in distantly related organisms. Some authors have interpreted this observation as implying that genome plasticity results in a more or less random gene order (Dobrindt *et al.*, 2002) rather than in rules of organization. However, even in the case of the most plastic genomes, those of Cyanobacteria, remaining islands of cluster conservation are still observed (Fang *et al.*, 2005, 2008; Shi & Falkowski, 2008). Specific clustering processes need to be identified to account for this.

Three main hypotheses have been proposed to account for gene clustering in bacterial genomes: (1) clusters result from local gene duplication followed by divergence; (2) genes display ‘selfish’ behaviour, aggregating into clusters to increase their chances of propagating through horizontal transfer into other genomes; and (3) selective advantages induce clustering in chromosomes (Fang *et al.*, 2008).

The first hypothesis for gene clustering fits well with acquisitive evolution at the origin of metabolic pathways: enzymes in the pathway may derive from related polypeptides because they work on related substrates (see Danchin, 1989 for a discussion). This is indeed observed in many metabolic operons. However, because genes enter and leave genomes frequently, local duplications cannot be the general cause of clustering. Analysis of biosynthetic operons in metabolic pathways shows that genes are sometimes grouped and sometimes spread out (Shi & Falkowski, 2008), while their order within an operon may be shuffled in different genomes (Parsot, 1986). This hypothesis cannot, therefore, be retained as a major cause of clustering.

A model presented by Lawrence & Roth (1996) attempted to substantiate the second hypothesis. The core assumption of their model was that because the consistency of a metabolic pathway is usually the result of the action of several genes, physical proximity is strongly correlated with clustering of cooperative functions. As physical proximity affects the probability of acquiring a global function, the operon and all genes associated with it will display selfish behaviour. This model would account for the presence in a population of a function that would be weakly selected in its own right, explaining why it avoids extinction. While exploring their model, the authors remarked that genes involved in essential processes should *not* tend to cluster together (Lawrence & Roth, 1996). This remark is important in the present context, as this would be diametrically opposed to the hypothesis we have defended. But as we have seen, the analysis of gene persistence contradicts Lawrence and Roth's prediction (Danchin *et al.*, 2007; Fang *et al.*, 2008).

The third hypothesis is by nature teleological [and therefore somewhat foreign to the standards of scientific reasoning; see, however, Allen *et al.* (1998)], but nevertheless it is very often used. The selective advantages of clustering have generally been discussed along two major lines: cotranscription of genes and functional coupling mediated by protein–protein interactions. A role for cotranscription, which is at the core of the concept of the operon, is supported by the observation that the functions of genes present in most operons are usually related to one another. Indeed, the very fact that genes tend to stay in a similar operon in widely different genomes has often been used to infer functional correlations (Overbeek *et al.*, 1999; Rogozin *et al.*, 2002), sometimes quite unexpected ones (Nitschké*et al.*, 1998; Noria & Danchin, 2002). In the same way, networks of protein interactions have been thought to play a considerable role in gene clustering in bacterial genomes, and these interactions form the core of many systems biology approaches (Arifuzzaman *et al.*, 2006; Baker *et al.*, 2006;Tamames *et al.*, 2007).

These interpretations, however, rely on a surprising underlying hypothesis. Where would the force grouping genes together come from? Where would the knowledge that they are better cotranscribed be located? What force would tell proteins that they should interact? Many investigators (and journals) have thus unwittingly introduced a kind of ‘intelligent design’ into their explanation of what they observe, without considering the catastrophic sociological consequence of this lack of deep understanding (Cornish-Bowden & Cardenas, 2007; Ayala, 2008). Our first objective must be to discover the mechanism that produces gene clustering, without relying on any instructive principle. Interestingly, this is much easier that one might have thought. In a genetic system where genes can get in and out frequently while maintaining a fairly constant genome size, clustering is a fairly straightforward consequence of the contribution of genes to the overall fitness of the organism, whatever the function contributing to fitness (Fang *et al.*, 2008). In short, clustering precedes cotranscription and protein–protein interactions, which can then easily be understood as contributing to a selective stabilization process (Changeux *et al.*, 1973) that keeps these associations together and causes their apparent robustness (Fang *et al.*, 2008).

## Writing on the genome: adaptive mutations and editing

The OS model fits well with the part of the paleome that is devoted to the construction of the cell (anabolism) and to replication (Fang *et al.*, 2005; Danchin, 2007). This paleome gene subset (constructor and replicator) more or less completely overlaps the set of genes found to be essential for life (Kobayashi *et al.*, 2003; Sassetti *et al.*, 2003; Joyce *et al.*, 2006). However, the paleome includes a set of genes that are not essential for life under laboratory growth conditions (Fang *et al.*, 2005). Many of these genes code for maintenance and repair, and may be involved in perpetuating life by restoring accuracy and even creating information during the reproduction process (Danchin, 2008a). In this context, the Turing machine model of the cell provides us with a novel way to consider the constraints of evolution. Indeed, in this model, the machine can not only read the program, but it can also write on it (remember that there is no conceptual difference between data and program). It is therefore acceptable that, under particular circumstances, the genetic program itself is modified, a statement that sounds fairly heretical.

As previously commented, the existence of a remarkable category of mutations, ‘adaptive mutations’, has stirred the community. Emotions ran high not because they exist [and they have been observed repeatedly (Foster & Cairns, 1992; Danchin, 1993 (2007); Hall, 1998)], but because of the unfortunate Lamarckian stance some people have taken to account for their existence, suggesting that they are directed mutations (Cairns *et al.*, 1988; Danchin, 1988b; Rosenberg, 1997). In a study typical of a systems biology approach, Fong & Palsson (2004) demonstrated that consistency between metabolic organization and phenotype during adaptive evolution led to large increases in growth rate for gene-deletion strains, while the underlying characteristics of the mutants obtained independently differed widely. Interestingly, some of the mutator polymerases (PolIV and PolV) that could be responsible for adaptive mutations (Tompkins *et al.*, 2003) belong to the paleome (Danchin *et al.*, 2007). This is quite difficult to observe, as they evolve very fast, and the fact that they belong to the paleome means that the definition of persistent genes must be relaxed (Fang *et al.*, 2005, 2008).

The model of the cell as a computer making computers becomes particularly interesting at this point. Indeed, as we have stressed repeatedly, not only does it separate between the machine and the program, but it separates between two different duplication processes: one for the machine, reproduction, and one for the program, replication. As Dyson showed, reproduction can improve over time, while replication usually cannot (Dyson, 1985). Analysis of the paleome has suggested that a substantial proportion of its genes are devoted to coding for functions involved in a ratchet-like accumulation of information (Danchin, 2008a). The involvement of ‘unfaithful’ DNA polymerases in producing adaptive mutations (Rosenberg, 1997) substantiates the importance of a constructive feedback mechanism that would couple reproduction to replication in the following way. Alterations of the replicated DNA, resulting either from the direct action of DNA polymerases, or from the indirect effect of transcription (Wright, 2004), would be triggered when cells face a situation in which there is no predictable outcome, except death (Rosenberg, 1997). Under such circumstances, an energy-driven, selective degradation process would make room for the accumulation of entities that remain functional (Danchin, 2008a). This coupling between reproduction and replication gives further weight to the Turing machine model of the cell, and opens a novel avenue to explore the evolution of living organisms. Systems biology models are needed to explore analytically the domain of application of this coupling.

Finally, I would like to speculate on a puzzling feature of bacterial genomes that may have a role in the process of accumulating information. In general, the A+T content of the genome is not uniform, with some regions particularly A+T-rich. This has been explained by horizontal gene transfer coupled to a systematic bias against incorporation of C into genomes, because of the way pyrimidine is constructed, in relation to the way deoxyribonucleotides are synthesized (Cohen, 1960; Nitschké*et al.*, 1998; Noria & Danchin, 2002; Rocha & Danchin, 2002). However, some genomes have G+C-rich islands (Muller *et al.*, 2007), and some genomes are enriched in G+C overall (Streptomycetes and Myxobacteria, for example). This type of nucleotide enrichment requires other explanations or complementary ones. In higher eukaryotes a family of proteins is involved in the fight against viral infection by systematically altering viral genomes. APOBEC proteins deaminate cytosines locally in RNA (Holmes *et al.*, 2007) and ADAR proteins deaminate adenines locally (Valente & Nishikura, 2005). If similar processes could operate in Bacteria and play a role on chromosomal DNA, directly or indirectly, one could expect to find local enrichment of the genome in A+T in the case of cytosine deaminases and G+C in the case of adenine deaminases. Genes belonging to these families exist in many bacterial genomes. They have always been thought to be involved in scavenging nucleic bases, nucleosides or nucleotides. A possible contribution to the evolution of the genetic program, triggered by some fight against virus infection and permitted by the Turing machine model, seems worth investigating.

## Conclusion

The use a cell of a given species as a recipient for the genome of another species has extended the previous remarkable feat of the cloning of the ewe Dolly. Conceptually, this remarkable experiment lends substance to the image of the cell as a computer. The physical separation in the cell between the cell machinery, which can reproduce, and the chromosome, which replicates, means that the cell can be seen as a kind of Turing machine, a computer. In this frame of thought, the program is not different from the data carried by the tape read by the machine. This implies that the role of what we term the ‘program’ is purely *declarative*. It does not need instructions: the presence of the tape carrying the program in the machine is enough to trigger the process of reading and deciphering its message, followed by changes of states in the machine and associated actions. (While the word ‘system’ is remarkably vague, and ‘synthetic’ emphasises the role of artifice in the construction of cells, it may be better to stress the role of integration in the new trends of biology. The work ‘symplectic’ constructed from the Greek, πλɛκτɛιν, to weave, and συν, together, would be more appropriate (de Lorenzo & Danchin, 2008). This is the more so because this word has no connotation associated with it, which would prevent intrusion of irrational discussions in a purely scientific context.)The most important prediction of this model, perhaps, is that it sees life as a process that enables material systems to manipulate, create and accumulate information. And, using information as an authentic category of Nature (alongside matter, energy, space and time), this is achieved without resorting to any principle other than those on which physics is based – a point of no small importance at a time when, curiously, some people wish to regress to an age when humans desperately needed to believe in external principles, to accept their life on Earth.

As science progresses, there is, in parallel, a steady decrease in the number of postulates on which it has to rely for its development. A common objection to the view of the cell as a computer is based on the physical nature of DNA, which has other roles besides carrying the genetic program. DNA sequences can play the role of spacers or of timers. Yet it must be accepted that when the Turing Machine has to be constructed as a concrete physical entity – a computer – the program running the machine needs a physical support. A punched tape, a magnetic disk, a CD or a flash memory are completely different materials. This has no influence on the conceptual nature of the program in the machine (of course, it has considerable influence on the physical nature of the computer!). Hence, the objection does not hold. However, this means that the physical state of the program may be important. This is where epigenetics begins.

## Statement

Reuse of this article is permitted in accordance with the Creative Commons Deed, Attribution 2.5, which does not permit commercial exploitation.
